# Determinants of knowledge of the highest conception probability period among reproductive age women in Low-Income African countries: A multilevel analysis based on the recent Demographic and Health Survey

**DOI:** 10.1371/journal.pone.0287164

**Published:** 2023-06-15

**Authors:** Mengistie Diress, Daniel Gashaneh Belay, Mohammed Abdu Seid, Habitu Birhan Eshetu, Anteneh Ayelign Kibret, Dagmawi Chilot, Mihret Melese, Deresse Sinamaw, Wudneh Simegn, Abdulwase Mohammed Seid, Amare Agmas Andualem, Desalegn Anmut Bitew, Yibeltal Yismaw Gela

**Affiliations:** 1 Department of Human Physiology, School of Medicine, College of Medicine and Health Sciences, University of Gondar, Gondar, Ethiopia; 2 Department of Human Anatomy, School of Medicine, College of Medicine and Health Sciences, University of Gondar, Gondar, Ethiopia; 3 Department of Epidemiology and biostatistics, Institute of Public Health, University of Gondar, Gondar, Ethiopia; 4 Unit of Human Physiology, Department of Biomedical Science, College of Health Sciences, Debre Tabor University, Debre Tabor, Ethiopia; 5 Department of Health Education and Behavioral Sciences, Institute of Public Health, University of Gondar, Gondar, Ethiopia; 6 Addis Ababa university, college of health sciences, Center for innovative Drug Development and Therapeutic Trials for Africa (CDT-Africa), Addis Ababa, Ethiopia; 7 Unit of Human Physiology, Department of Biomedical Science, College of Health Sciences, Debre Markos University, Debre Tabor, Ethiopia; 8 Department of social and administrative Pharmacy, School of Pharmacy, College of Medicine and Health Sciences, University of Gondar, Gondar, Ethiopia; 9 Department of Clinical Pharmacy, School of Pharmacy, College of Medicine and Health Sciences, University of Gondar, Gondar, Ethiopia; 10 Department of Anesthesia, Wollo University, Dessie, Ethiopia; 11 Department of Reproductive Health, College of Medicine and Health Sciences, University of Gondar, Gondar, Ethiopia; Jawaharlal Nehru University, INDIA

## Abstract

**Background:**

Adequate knowledge about the highest conception probability period in the reproductive cycle allows individuals and couples to attain or avoid their fertility probabilities. Poor knowledge of conception probability period leads to undesirable outcomes like unwanted pregnancy, miscarriage, and abortion. Determinants of knowledge of highest conception probability period were not well studied on economically disadvantaged countries. Therefore, our study aimed to identify individual and community level factors of knowledge of the highest conception probability period among women of reproductive age in low income African countries.

**Methods:**

The appended and latest Demographic and Health Survey datasets of 15 low-income African countries was used for analysis. Model fitness was done using the Intraclass correlation coefficient, median odds ratio, and deviance. A model with the lowest deviance (model-III) was selected as the best model. Multilevel logistic regression model was used to identify determinant factors of knowledge of the highest conception probability period. In the final model, adjusted odds ratio with 95% confidence interval was reported and variables with p<0.05 were considered as statistically significant with knowledge of the highest conception probability period.

**Results:**

Total weighted sample of 235,574 reproductive aged women with a median age of 27 years were included. The correct knowledge of the highest conception probability period among the study participants was 24.04% (95%CI = 23.87–24.22%). Maternal age groups ((20–24 (AOR = 1.49; 95%CI = 1.44–1.55), 25–29 (AOR = 1.62; 1.56–1.68), 35–39 (AOR = 1.76; 1.68–1.84), 40–44 (AOR = 1.75; 1.67–1.83), and 45–49 (AOR = 1.83; 1.74–1.93)), marital status((currently in union (AOR = 1.75; 1.16; 1.13–1.20), formerly in union (AOR = 1.75; 1.11; 1.06–1.16)), better educational status ((secondary (AOR = 2.08; 2.01–2.14) and higher(AOR = 3.36; 3.18–3.55)), higher wealth index ((middle (AOR = 1.08; 1.04–1.12), richer (AOR = 1.24; 1.20–1.28), and richest (AOR = 1.51; 1.45–1.57)), knowledge of contraceptive methods (AOR = 2.63; 2.49–2.77), current contraceptive use (AOR = 1.14; 1.11–1.16), and urban residency (AOR = 1.26; 1.21–1.29) were statistically significant with knowledge of the highest conception probability period.

**Conclusion:**

In this study, knowledge of the highest conception probability period among women of reproductive age in low-income African countries was low. Therefore, improving the fertility awareness through comprehensive reproductive education or counseling could be one of the operational ways to control unintended pregnancy.

## Introduction

Female reproductive cycle is a physiological process which starts in girls at puberty. This incident involves both the uterine (bleeding, proliferative & secretory phases) and ovarian (pre-ovulatory, ovulation & post-ovulatory phases) cycles [1]. Ovulation is an event where the dominant follicle releases its egg from the ovary into the fallopian tube for potential fertilization and is regulated by a surge in the luteinizing hormone [1, 2]. Critical knowledge when the women’s menstrual period starts, when they ovulate and when their next menstrual period dues are significant records for them to reduce the risk of unwanted or unplanned pregnancy. Therefore, awareness about the time of ovulation (detected by basal body temperature and cervical mucus) can be used as a natural contraceptive method because it helps them to estimate when the highest conception probability period is [3, 4]. Being aware of one’s highest conception probability period of the reproductive cycle is basic to realize fertility probabilities and a number of women in the world used it as family planning method [5–7].

A study conducted in 2016 and 2013 in USA revealed that about 32.8% and 60% of women know the timing of ovulation respectively [5, 8]. Based on a study in India, the prevalence of correct knowledge of the highest conception probability period among women is 15% [9]. A study in 29 African countries discovered that, the prevalence of knowledge of ovulation ranged from 10.4%-49% [10]. Another study conducted in Ghana showed that about 38% of women have knowledge on the mid period of ovulation in their reproductive cycle [11]. Two studies in Ethiopia reported that the correct knowledge of ovulatory cycle among women is about 23.6% [12, 13].

Different studies on knowledge of female reproductive cycles in the world have shown that women’s age, education, marital status, place of residence, media exposure, and knowledge and current usage of contraceptive methods are common factors associated with knowledge of the highest conception probability period [5, 8, 9, 11, 14–17]. Based on a recent study in Sub-Saharan countries, correct knowledge of ovulation timing is strongly affected by the low socioeconomic status of the women and leads to unplanned pregnancy [18].

Even though many studies showed the association between knowledge of ovulation timing and economic status of women in reproductive age, other individual and community level determinants of knowledge of the highest conception probability period were not well studied in economically disadvantaged countries. Therefore, this study aimed to determine the prevalence and identify determinant factors of correct knowledge of the highest conception probability period among reproductive-age women in low-income African countries using the Demographic and Health Survey datasets at the individual and community levels.

## Methods

### Data source, study design, areas, and period

We used the appended and latest nationally representative Demographic and Health Survey (DHS) datasets of 15 low-income African countries after a reasonable request from the Measure DHS programme [19] available at https://dhsprogram.com/Data/terms-of-use.cfm. In this study, we used the standard DHS data set for each country to have all parameters and a large sample size which can be generalizable for the source of population. The standard DHS survey of each country was a community-based cross-sectional study conducted every five year interval and provides the updated health and health-related indicators at the national and sub-national level.

### Population and sample size

The source populations were all women of reproductive age (15–49 years) across low-income African countries and those living in the selected enumeration areas (EAs) were the study population. All women aged 15–49 years in the selected EAs in each country were included in this study. We used women’s dataset (IR file) and the total weighted samples of 235,574 women for analysis.

### Sampling procedure

Based on the recent World Bank lists of economies, 23 African countries are grouped under low-income level (Burundi, Burkina Faso, Central African Republic, Congo, Dem. Rep, Eritrea, Ethiopia, Guinea, Gambia, Guinea‐Bissau, Liberia, Madagascar, Mali, Mozambique, Malawi, Niger, Rwanda, Sudan, Sierra Leone, Somalia, South Sudan, Chad, Togo, and Uganda). Of these countries, only fifteen low-income African countries (Burundi, Congo Dem. Rep, Ethiopia, Guinea, Gambia, Liberia, Mali, Mozambique, Malawi, Niger, Rwanda, Sierra Leone, Chad, Togo, and Uganda) were included in this study. However, eight countries were excluded due to the unavailability of DHS dataset publicly.

### Data collection

Pre-tested, structured, and interviewer-administered standard DHS questionnaires were used for data collection of the DHS surveys. The questionnaire was conceptualized to the different countries context and the data were collected by trained data collectors. The questionnaire was prepared in English and then translated to the local languages [20].

### Study variables

The outcome variable of the current study was knowledge of the highest conception probability period among women of reproductive age during their ovulatory cycle. The DHS surveys collected data about knowledge of the highest conception probability period on women by asking their ovulation timing. The possible responses of the women were labelled as: "1 = during her period", "2 = after period ended", "3 = middle of the cycle", "4 = before the period begins", "5 = at any time", "6 = other", and "7 = don’t know" [21]. The correct knowledge of the highest conception probability period was defined as the “middle of the cycle”.

In this study, both individual and community-level independent variables have been studied. Individual-level determinant factors for knowledge of the highest conception probability period were assessed on the women’s age, wealth index, media exposure, highest level of education, marital status, menstruation status in last six weeks, knowledge of any contraceptive methods, current use of contraceptive methods, and information about family planning methods on media. Media exposure status of the women was created from the frequency of watching television, listening to the radio, and reading a newspaper or magazine. If a woman has at least one “yes”, then she was considered as having media exposure.

The community-level variables include: region of the country in Africa, type of residence, DHS survey year, community-level media exposure, and community-level education. Community-level media exposure was created by summing up of all the "yes" in each category from the women’s exposure in television, newspaper/magazine, and radio. Then, the proportion of media exposure was categorized as high (when greater or equal to median) and low (when lower than the median). Community-level education was created by considering those women with no education as "no" and education level of primary, secondary and higher categorized as “Yes”. Then, the proportion was calculated from the "yes" education group and it was coded as “0” for low (communities in which < 50% women had at least primary education) and “1” for high community-level women education (communities in which ≥ 50% women had at least primary education) at cluster level.

### Data management and statistical analysis

After DHS dataset were downloaded in STATA format, we had cleaned, integrate, transformed, and appended the data to have appropriate variables for the analysis. Stata version 16 and Microsoft excel were used for extraction, recoding, and for generating both descriptive and analytic statistics of the appended 15 countries’ data. Prior to statistical analysis, the data were weighted (using women’s sampling weight) to ensure the representativeness of the DHS sample and to get consistent statistical estimates. The DHS data has hierarchical nature, and women aged 15–49 years were within a cluster. This might violate the standard logistic regression model assumptions. Therefore, a multilevel logistic regression model was fitted. Four models were fitted for multi-level analysis. The first was the null model containing only the outcome variable which was used to check the variability of knowledge of the highest conception probability period across the cluster. The other fitted models with the prevalence of knowledge of the highest conception probability period were; model I (containing individual-level factors only), model II (containing community-level factors only), and model III (both individual- and community-level factors were fitted simultaneously). Model comparisons were done using the deviance test and log likelihood test and the model with the highest log likelihood ratio and the lowest deviance was selected as the best-fitted model. Therefore, Model III was the best-fitted model for this data since it had the lowest deviance value compared to the other models. We have estimated the random effects (a measure of variation) using Intra-class Correlation Coefficient (ICC), Median Odds Ratio (MOR), and Proportional Change in Variance (PCV) which were used to check the need for multilevel logistic regression analysis. A multilevel binary logistic regression analysis was performed to identify individual and community level determinant factors of knowledge of the highest conception probability period. Variables with a p-value ≤ 0.2 in the bivariable multilevel binary logistic regression have been selected as candidates for the final model. Crude Odds Ratio (COR) and the Adjusted Odds Ratio (AOR) were assessed and finally adjusted odds ratio (AOR) were presented. In the multivariable multilevel logistic regression, independent variables with a p-value of < 0.05 were considered as statistically significant factors of the correct knowledge of the highest conception probability period The strength and direction of the association between the correct knowledge of the highest conception probability period and independent variables has been reported using Adjusted Odds Ratio (AOR) along with its 95% Confidence Interval (CI).

### Ethical approval

We obtained online permission from the Demographic and Health Surveys (DHS) program data archivists to download the dataset for this study. The dataset was kept in private to maintain its confidentiality.

## Results

### Characteristics of the study participants

A total weighted samples of 235,574 reproductive aged women with a median age of 27 (IQR = 20–35) years were included in this study. About Twenty-two percent of the respondents were aged 15–19 years. About half of the women (51.65%) were from East Africa. Most of the women (70.02%) were rural dwellers and more than three-fifths of women (64.2%) had formal education. About three-fifths of women (61.10%) had media exposure and Ninety-three percent of the study participants had self-reported knowledge about any type of contraceptive methods. However, about seventy-eight percent of the women did not use contraceptive methods currently (Table 1).

**Table 1:** Sociodemographic and economic characteristics of respondents knowledge of the highest conception probability period (n = 235,575/4).

Variables	Categories	Frequency (n)		Weighted Percentage (%)
		Unweighted	Weighted	
Age (years) in 5 years groups	15–19	51,455	50,853	21.59
	20–24	43,094	43,448	18.44
	25–29	40,656	41,298	17.53
	30–34	33,462	33,709	14.31
	35–39	29,055	28,915	12.27
	40–44	20,861	20,563	8.73
	45–49	16,992	16,787	7.13
Regions in African countries	Central Africa	36,382	36,381	15.44
	East Africa	121,668	121,668	51.65
	West Africa	77,525	77,525	32.91
DHS survey year	< = 2015	121,816	121,815	51.71
	>2015	113,759	113,759	48.29
Residence	Urban	73,537	70,632	29.98
	Rural	162,038	164,942	70.02
Marital status	Never in union	62,043	61,897	26.28
	Currently in union	152,859	153,007	64.95
	Formerly in union	20,673	20,670	8.77
Educational level of women	No education	85,938	84,300	35.79
	Primary	83,598	84,248	35.76
	Secondary	58,300	58,800	24.96
	Higher	7,739	8,226	3.49
Wealth index	Poorest	46,812	43,027	18.26
	Poorer	42,878	44,289	18.80
	Middle	43,543	45,028	19.11
	Richer	45,349	47,803	20.29
	Richest	56,993	55,427	23.53
Women’s media exposure	No	93,153	91,628	38.90
	Yes	142,422	143,946	61.10
Heard family planning on television last few months	No	207,637	209,045	88.75
	Yes	27,906	26,496	11.25
Heard family planning on newspaper or magazine last few months	No	223,672	224,022	95.12
	Yes	11,853	11,498	4.88
Heard family planning on radio last few months	No	159,739	159,275	67.62
	Yes	75,808	76,270	32.38
Community level media exposure	Low	117,964	117,386	49.83
	High	117,611	118,188	50.17
Community level of education	Low	117,982	114,621	48.66
	High	117,593	120,953	51.34
Menstruated in last six weeks	No	88,915	89,764	38.10
	Yes	146,660	145,810	61.90
Knowledge of any contraceptive methods	No	18,202	15,369	6.52
	Yes	217,373	220,205	93.48
Current contraceptive use	No	184,281	182,936	77.66
	Yes	51,294	52,638	22.34

### Knowledge of the highest conception probability period among women of reproductive age

**Fig 1:**
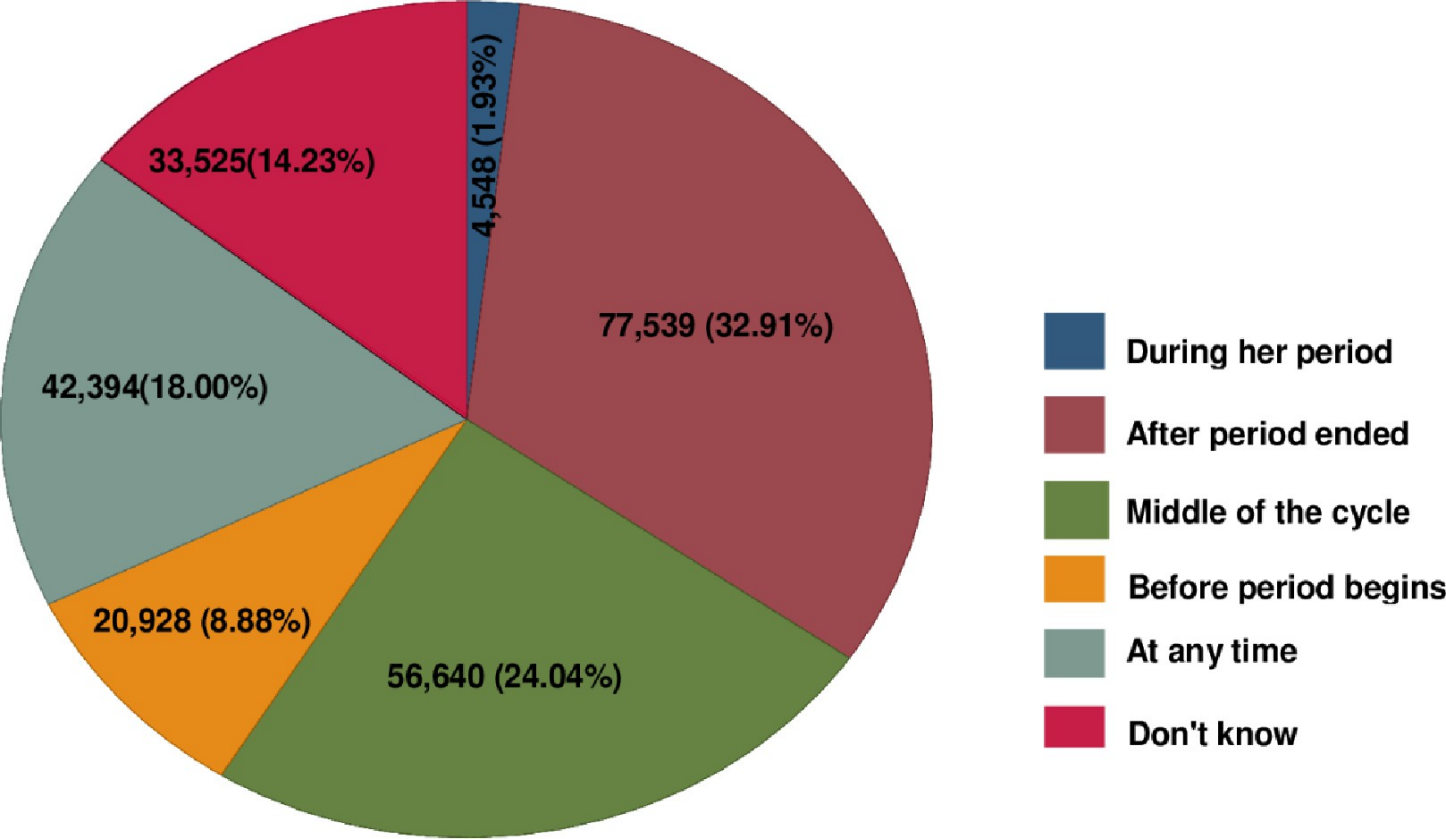
A pie chart depicting the distribution of reproductive age women with their knowledge of the ovulatory cycle in Low-Income African countries based on the recent Demographic and Health Surveys.

From the total weighted study participants drawn from 15 low-income African countries, 24.04% (95% CI: 23.87–24.22%) of them had correctly responded for the highest conception probability period (middle of the cycle). In contrast, 178,933 (75.96%) women had incorrectly responded about knowledge of the highest conception probability period. The percentage of respondents who replied as the ovulation timing is “after period ended” was 32.91% (Fig 1).

### Random effects analysis and model comparison

As shown in Table 2, the Intra-class Correlation Coefficient (ICC) in the null model was 10.13%, indicating that about 10% of the variations in knowledge of the highest conception probability period among women were attributed to cluster differences, whereas the remaining 90% were attributed to individual women factors. The Median Odds Ratio (MOR) value of 1.78 in the null model revealed that the odds of responding the correct the highest conception probability period among study subjects were significantly different between clusters. Moreover, the Proportional Change in Variance (PCV) value, 43.67% in the final model (model-III), indicates that there was about 44% variation in knowledge of the highest conception probability period among study subjects. For model comparison/fitness, deviance was used. The model with the lowest deviance was the better fitted model, which was model-III in this study (Table 2).

**Table 2:** Random effect analysis and model comparison results.

Parameter	Null model	Model-I	Model-II	Model-III
Community-level variance	0.371 (0.325–0.394)	0.239 (0.221–0.260)	0.214 (0.200–0.231)	0.209(0.195–0.226)
ICC (%)	10.13 (9.65–10.74)	6.77(6.16–7.04)	6.11(5.94–6.83)	5.97(5.42–6.68)
MOR	1.78(1.40–1.97)	1.59(1.39–1.86)	1.55(1.35–1.81)	1.54(1.34–1.80)
PCV (%)	Ref	35.58	42.32	43.67
Log-likelihood	-128307.86	-123468.98	-123860.96	-119191.4800
Deviance	256615.72	246937.96	247721.92	238382.96
LR test	X^2^ = 3261.34, p<0.001	X^2^ = 2877.64, p<0.001	X^2^ = 2554.52, p<0.001	X^2^ = 2332.40, p<0.001

ICC = Inter cluster correlation coefficient, MOR = Median odds ratio, PCV = proportional change in variance

### Factors associated with knowledge of the highest conception probability period among women of reproductive age in low-income African countries

All individual and community level variables had a p-value < 0.20 in the bivariable multilevel logistic regression analysis and eligible for multivariable analysis. Then, in multivariable multilevel logistic regression analysis, variables were analyzed in four models. These were the null model with only dependent variable, model-I with only individual level predictor variables, model-II with only community level predictor variables and the final model (model-III) with both individual level and community level variables run simultaneously. Finally, the AOR with 95% CI and p-value < 0.05 in the final model was reported.

Based on the multivariable multilevel logistic regression analysis in the final model, age of women in years, maternal educational status, marital status, place of residence, wealth index, regions in African countries, knowledge of contraceptive methods, current use of contraceptives, menstruation in the last six weeks, heard family planning methods on newspaper or magazine last few months, and DHS survey year were found to be significantly associated with correct knowledge of the highest conception probability period (p<0.05).

Women aged 20–24, 25–29, 30–34, 35–39, 40–44, and 45–49 were 1.49 (95%CI = 1.44–1.55), 1.62 (95%CI = 1.56–1.68), 1.68 (95%CI = 1.61–1.75), 1.76 (95%CI = 1.68–1.84), 1.75 (95%CI = 1.67–1.83), and 1.83 (95%CI = 1.74–1.93) times more likely to have correct knowledge of the highest conception probability period than those aged 15–19 years, respectively. Those women currently and formerly in union were 1.16 (95%CI = 1.13–1.20) and 1.11 (95%CI = 1.06–1.16) times more likely to have correct knowledge of the highest conception probability period respectively than women with never in union. The odds of having correct knowledge of the highest conception probability period was 1.22 (95%CI = 1.19–1.26), 2.08 (95%CI = 2.01–2.14) and 3.36 (95%CI = 3.18–3.55) times better among women who had primary, secondary and higher educational levels respectively than those who did not have formal education. The odds of responding the correct knowledge of the highest conception probability period among women from middle, richer, and richest households was increased by 8% (AOR = 1.08; 95%CI = 1.04–1.12), 24% (AOR = 1.24; 95%CI = 1.20–1.28), and 51% (AOR = 1.51; 95%CI = 1.45–1.57) respectively as compared to women from poorest households. Women who heard about family planning methods on newspaper or magazine in last few months were 1.13(95%CI = 1.07–1.18) times more likely to have correct knowledge of the highest conception probability period than the counterparts. Women who menstruated in the last six weeks had 1.08(95%CI = 1.06–1.11) times better knowledge of the highest conception probability period than the counterparts. The odds of correct knowledge of the highest conception probability period was 2.63 (95%CI = 2.49–2.77) times more likely in the women with self-reported knowledge of contraceptive methods than those who did not know about contraceptives. Correct knowledge of the highest conception probability period was increased by 14% (AOR = 1.14; 95%CI = 1.11–1.16) among the women who currently used contraceptives than those who did not use. Women from East Africa and West Africa were 79% (AOR = 0.21; 95%CI = 0.20–0.22) and 60% (AOR = 0.40; 95%CI = 0.39–0.42) times less likely to have correct knowledge of the highest conception probability period respectively than those who were from Central Africa. The correct knowledge of the highest conception probability period was increased by 59% (AOR = 1.59; 95%CI = 1.55–1.63) among the women whose DHS survey year is after 2015. Finally, the odds of correct knowledge of the highest conception probability period among the women was 1.26 (1.21–1.29) times higher in urban dwellers than in rural residents (Table 3).

**Table 3:** Multilevel logistic regression analysis of determinant factors of knowledge of the highest conception probability period among women of reproductive age in Low-Income African countries (n = 235,574).

Variables	Categories	Knowledge of conception period (weighted)		AOR (95% CI)		
				Model I	Model II	Model III
		No N (%)	Yes N (%)			
Age (years) in 5 years groups	15–19	41,852(82.30)	9,001(17.70)	1	__	1
	20–24	32,270(74.27)	11,178(25.73)	1.46(1.41–1.51)***	__	1.49(1.44–1.55)***
	25–29	30,239(73.22)	11,059(26.78)	1.60(1.54–1.66)***	__	1.62(1.56–1.68)***
	30–34	24,997(74.15)	8,712(25.85)	1.61(1.55–1.68)***	__	1.68(1.61–1.75)***
	35–39	21,458(74.21)	7,457(25.79)	1.69(1.62–1.76)***	__	1.76(1.68–1.84)***
	40–44	15,434(75.06)	5,129(24.94)	1.66(1.58–1.73)***	__	1.75(1.67–1.83)***
	45–49	12,684(75.56)	4,103(24.44)	1.72(1.63–1.80)***	__	1.83(1.74–1.93)***
Marital status	Never in Union	48,273(77.99)	13,624(22.01)	1	__	1
	Currently in union	114,806(75.03)	38,201(24.97)	1.31(1.27–1.35)***	__	1.16(1.13–1.20)***
	Formerly in union	15,854(76.70)	4,815(23.30)	1.13(1.08–1.18)***	__	1.11(1.06–1.16)***
Educational level of women	No education	66,906(79.37)	17,394(20.63)	1	__	1
	Primary	68,050(80.77)	16,198(19.23)	1.00(0.97–1.02)	__	1.22(1.19–1.26)***
	Secondary	39,670(67.47)	19,130(32.53)	2.10(2.04–2.16)***	__	2.08(2.01–2.14)***
	Higher	4,308(52.37)	3,918(47.63)	3.23(3.06–3.41)***	__	3.36(3.18–3.55)***
Wealth index	Poorest	34,810(80.90)	8,217(19.10)	1	__	1
	Poorer	35,312(79.73)	8,977(20.27)	1.05(1.02–1.09)*	__	1.03(0.99–1.07)
	Middle	35,368(78.54)	9,661(21.46)	1.10(1.07–1.14)***	__	1.08(1.04–1.12)***
	Richer	35,920(75.14)	11,883(24.86)	1.25(1.20–1.29)***	__	1.24(1.20–1.28)***
	Richest	37,524(67.70)	17,903(32.30)	1.46(1.40–1.51)***	__	1.51(1.45–1.57)***
Women’s media exposure	No	71,618(78.16)	20,010(21.84)	1	__	1
	Yes	107,315(74.55)	36,631(25.45)	0.92(0.90–0.94)***	__	1.00(0.97–1.02)
Heard FP on television last few months	No	160,568(76.81)	48,477(23.19)	1	__	1
	Yes	18,346(69.24)	8,496(30.76)	1.00(0.97–1.04)	__	1.00(0.96–1.03)
Heard FP on newspaper or magazine last few months	No	171,034(76.35)	52,988(23.65)	1	__	1
	Yes	7,868(68.42)	3,630(31.58)	0.99(0.95–1.04)	__	1.13(1.07–1.18)***
Heard FP on radio last few months	No	121,186(76.09)	38,089(23.91)	1	__	1
	Yes	57,731(75.69)	18,539(24.31)	0.85(0.83–0.87)**	__	1.01(0.99–1.04)
Menstruated in last six months	No	69,501(77.43)	20,263(22.57)	1	__	1
	Yes	109,432(75.05)	36,378(24.95)	1.09(1.07–1.11)***	__	1.08(1.06–1.11)***
Knowledge of any contraceptive methods	No	13,375(87.02)	1,994(12.98)	1	__	1
	Yes	165,559(75.18)	54,646(24.82)	1.71(1.63–1.80)***	__	2.63(2.49–2.77)***
Current contraceptive use	No	140,263(76.67)	42,673(23.33)	1	__	1
	Yes	38,671(73.47)	13,967(26.53)	0.99(0.97–1.01)	__	1.14(1.11–1.16)***
Regions in African countries	Central Africa	22,442(61.69)	13,939(38.31)	__	1	1
	East Africa	99,904(82.11)	21,764(17.89)	__	0.28(0.27–0.29)***	0.21(0.20–0.22)***
	West Africa	56,588(72.99)	20,937(27.01)	__	0.48(0.43–0.46)***	0.40(0.39–0.42)***
DHS survey year	< = 2015	92,865(76.23)	28,950(23.77)	__	1	1
	>2015	86,068(75.66)	27,691(24.34)	__	1.54(1.51–1.58)***	1.59(1.55–1.63)***
Residence	Urban	49,065(69.47)	21,567(30.53)	__	1.53(1.49–1.56)***	1.26(1.21–1.29)***
	Rural	129,868(78.74)	35,074(21.26)	__	1	1
Community level media exposure	Low	90,154(76.80)	27,232(23.20)	__	1	1
	High	88,780(75.12)	29,408(24.88)	__	1.06(1.00–1.15)	1.01(0.96–1.07)
Community level of education	Low	87,974(76.75)	26,647(23.25)	__	1	1
	High	90,959(75.20)	29,994(24.80)	__	1.04(0.99–1.58)	0.97(0.92–1.02)

## Discussion

Better knowledge about the highest conception probability period in the reproductive cycle allows individuals and couples to anticipate and attain their pregnancy or to avoid unintended pregnancy [22, 23]. Lack of knowledge about the highest conception probability period among the women will lead to lots of undesirable outcomes such as unwanted pregnancy, unplanned childbearing, miscarriage, and abortion especially for those women living in low-income developing countries [10, 18, 24, 25]. Hence, this study aimed to determine the correct knowledge of the highest conception probability period and identify the determinant factors among women of reproductive age in low-income African countries.

In this study, the correct knowledge of the highest conception probability period was 24.04% (95% CI: 23.87–24.22%) which is higher than the previous studies in India (15%) [9], Sub-Saharan Africa (8.3%) [18], Namibia (13.9%) [10], Nigeria (20.3%) [10], Zambia (21.5%) [10], Rwanda (21%)) [10], Kenya (23.4%) [10], and Ethiopia (23.6%) [12, 13]. This variation might be accounted due to differences sample size and sociocultural factors (lifestyles, religion, beliefs, values, social classes, sexuality and attitudes) among the study participants. On the other hand, our finding in this study is lower than studies conducted in USA (32.8%) [5], Comoros (49%) [10], Ghana (38%) [11], Togo (42.8%) [10], and Sierra Leone (30.3%)) [10]. This dissimilarity might be as a result of variations in study settings and socioeconomic status of the women in different countries. For instance, women with lower socioeconomic status have less access to economy, education, and health services than those with a higher socioeconomic status).

In multivariable multilevel logistic regression analysis, both individual and community-level variables were found to be associated with knowledge of the highest conception probability period. In our study, there is a significant difference in knowledge of the highest conception probability period among different age categories of women. Women in the advanced age groups were found to be more knowledgeable than women in the early age group (15–19 years). This result is in line with studies in Bangladesh [15], Spain [16], Pakistan [26], Ghana [11], USA [8], and Ethiopia [12, 13]. The possible reason behind for this association might be attributable to repetitive exposure to variety of experiences related to the concept of reproduction with increasing age or through life.

Marital status was another factor significantly associated with knowledge of the highest conception probability period. Women in union were more likely to have correct knowledge of the highest conception probability period than women who were not in any union which is supported by a study in Ghana [27]. The plausible association might be due to the programmed reminding roles of partners about the timing of menstrual cycle and ovulation dates.

Women who attained secondary and higher education had better knowledgeable about the highest conception probability period. This finding is similar with reports of many studies like in China [28], Sub-Saharan Africa and others [18, 23, 27, 29–31]. This result might be due to the fact that formal education provides better opportunities for the women to comprehend the science of reproductive system. Now a days, the impact of education on family planning methods including fertility awareness has been studied and approved that education increases the knowledge of individuals related to reproductive health [16, 23, 32]. Furthermore, it can be due to exposure in media like radio, television, newspaper or websites.

Economically privileged women (middle, richer, and richest wealth categories) were more knowledgeable about the highest conception probability period than women from poorest households. This result is in line with other studies in Sub-Saharan Africa [18] and Ethiopia [12]. This association might be as a result of good knowledge of women with advanced economic status [33]. Another reason could be also due to the presence of high desire for knowledge in people with higher wealth index [34].

Women who heard about family planning methods on a newspaper or magazine in the last few months were more knowledgeable about their highest conception probability period than those who had not heard. The possible reason for this association might be due to obtaining of information regarding of both the traditional and modern contraceptive methods through this media.

A better knowledge of the highest conception probability period was found in the women with self-reported knowledge of contraceptive methods than those who did not know about contraceptives. This finding is similar with studies in Ghana [11] and Malawi [35]. This might be attributable to an integration of the concept of ovulation in family planning counselling guidelines [36]. It might be also due to regular counselling of women about the significance of contraceptive methods including natural contraceptive practices (fertility awareness) [37, 38]. Usage of mass media and social networks also play chief roles in disseminating contraceptive knowledge [38].

Currently contraceptive users were found to have more knowledge of the highest conception probability period than those who did not in use which is in line with a study in Ethiopia [13]. This might be due to the good awareness of women about it.

Women who menstruated in the last six weeks had better knowledge of the highest conception probability period. This is similar with a study in Ethiopia [12, 13] and it might be because of recalling about their recent time of menstruation which in turn enables the women to consider when their ovulation and the highest conception probability period will be.

The result of our study demonstrates that knowledge of the highest conception probability period among the women was found to be higher in urban dwellers than in rural residents. This is in agreement with studies in China [28], Ethiopia [13], Africa [10], and Bangladesh [15]. The reason for having better knowledge in urban residents might be because of the favorable conditions like better state of affairs for socioeconomic and educational skills, increases access to media, internet/websites, and better utilization of health care services. Therefore, the cumulative effect provides a better information associated to family planning and other reproductive health services among urban residents.

Women from East Africa and West Africa had poor knowledge of the highest conception probability period as compared to those women from Central Africa. This might be due to differences in culture, life style, implementation of education, and political systems across different regions of Africa.

Knowledge of the highest conception probability period was better among the women whose DHS survey year is after 2015. The reason for this event can be explained in terms of time of the survey since the survey years of the countries included in our study ranges from 2011(Mozambique) to 2019(Liberia). Therefore, the recent the surveys may comprise many updated information about the women. Furthermore, due to globalization and increased access to education, respondents might have better knowledge about ovulation period.

### Strength and limitations of the study

Using large representative sample size (235,574) and applying appropriate statistical analysis (weighting and multilevel analysis) is a major strength of our study. It measures women’s knowledge of the highest conception probability period and determinant factors at individual and community levels in 15 low-income African countries and is believed to be generalizable to other developing countries. However, this study should be considered in light of some limitations. First, we used secondary data and some important independent variables like education levels of partners, the type of contraceptive use, regularity of the menstrual period, and nutritional status of the women were missed in the analysis. Second, DHS is cross-sectional in nature and hence we are unable to show the cause-effect relationship between dependent and independent variables. Lastly, since most of the collected data were based on self-report, social desirability and recall biases were not ruled out in this study.

## Conclusion

Most women in developing countries like Africa, used traditional and hormonal contraceptive methods which are associated with plenty of health risks on the women [39, 40]. Our study established that knowledge of the highest conception probability period among women of reproductive age in low-income African countries was low. Individual level factors (age, marital status, educational status, wealth index, currently using contraceptive methods, menstruated within the last 6 months, knowledge of contraceptive methods) and residence, regions in African countries and DHS survey year from community level factors were found to be significantly associated with knowledge of the highest conception probability period. Improving the fertility awareness through comprehensive reproductive education or counseling could be one of the operational ways to control unintended pregnancy. Future researchers are also recommend to address the missed independent variables using primary data.

## List of abbreviations

CI: Confidence interval
DHS: Demographic and Health Survey
ICC: Intraclass Correlation coefficient
MOR: Median Odds Ratio
PCV: Proportional Change in Variance
